# Regulatory roles of lncRNA *PANDAR* in breast cancer cell proliferation

**DOI:** 10.2478/abm-2021-0035

**Published:** 2021-12-30

**Authors:** Qinnuan Sun, Xiumei Wang

**Affiliations:** Department of Oncology, Inner Mongolia Cancer Hospital and Affiliated People's Hospital of Inner Mongolia Medical University, Hohhot, Inner Mongolia 010020, China; Medical Oncology, Affiliated People's Hospital of Inner Mongolia Medical University, Hohhot, Inner Mongolia 010020, China

**Keywords:** breast neoplasms, epithelial-mesenchymal transition, long noncoding RNA PANDAR, human, cell proliferation

## Abstract

**Background:**

Breast cancer represents the second most deadly malignancy in women, and long noncoding RNAs (lncRNAs) have crucial functions in its development.

**Objective:**

To investigate effects of the promoter of *CDKN1A* antisense DNA damage-activated RNA (*PANDAR*) on epithelial-mesenchymal transition (EMT) in breast cancer cells and their proliferation.

**Methods:**

lncRNAs potentially regulating the transcriptional activity of the E-cadherin (E-cad, an epithelial cell marker) gene promoter were screened using a dual-luciferase reporter assay. *PANDAR* was overexpressed in Michigan cancer foundation 7 (MCF-7) breast cancer cells. E-cad and N-cadherin (N-cad, a mesenchymal cell marker) levels were detected by immunoblotting. Cell viability was assessed using a cell counting kit-8.

**Results:**

*PANDAR* and *TCONS00068220*/*LOC105375819* conservatively regulated the promoter activity of *E-cad*. *PANDAR* overexpression in MCF-7 inhibited E-cad expression, but upregulated N-cad. The enhanced expression of *PANDAR* promoted cell proliferation.

**Conclusion:**

*PANDAR* is a key transcriptional repressor of *E-cad* and has regulatory effects on the promotion of cell proliferation. *PANDAR* is an oncogene in breast cancer, potentially facilitating the EMT process and promoting cell proliferation.

Breast cancer is a major malignancy affecting women worldwide [1]. Annually, approximately 1.4 million new breast cancer cases are recorded, with 410,000 women eventually dying, accounting for 14.1% of cancer-related deaths in female patients. Treatment for breast cancer has been reported to fail in 25%–40% of breast cancer patients within 5 years due to distant metastasis [2], which is the leading cause of death in affected individuals. Therefore, exploring the mechanisms that increase malignancy and poor treatment response is required to discover new biomarkers and treatment approaches.

Epithelial-mesenchymal transition (EMT) is a process by which epithelial cells undergo transformation to acquire mesenchymal features, generating malignant cells harboring stem cell-like properties, also known as cancer stem cells (CSCs), including migration and invasion, reduced apoptosis and senescence, and drug resistance [3], thus facilitating the metastatic cascade. A recent study found that the mesenchymal state is responsible for a lower degree of tumor cell differentiation, which is related to poor prognosis in patients [3]. EMT occurs throughout different stages of embryonic development and is induced via a panel of specific transcription factors. A previous review discussed the relationship between EMT-inducing transcription factors and cadherin modulation—these involving E-cadherin (E-cad) and N-cadherin (N-cad) through embryonic development and cancer progression [4]. A recent review indicated that a hallmark of EMT is the upregulation of N-cad followed by the downregulation of E-cad [5].

Long noncoding RNAs (lncRNAs) constitute a group of small RNAs with >200 bp and without an open reading frame, which inhibit the expression of downstream genes predominantly by affecting the activity of the upstream promoter region [6]. lncRNAs can contribute critically to the pathogenesis and development of breast cancer [7]. For example, the lncRNA *H19* promotes breast cancer cell proliferation, clone formation, and metastasis, and further generates CSCs [8]. However, studies assessing the regulation of the EMT process by lncRNAs in breast cancer are scarce. The promoter of the gene for cyclin dependent kinase inhibitor 1A, *CDKN1A* anti-sense DNA damage-activated RNA (*PANDAR*; NCBI gene ID: 101154753) is a noncoding RNA located on chromosome 6p21.2, whose abnormal expression is involved in the pathogeneses of various cancers [9, 10]. By inhibiting p16^INK4A^ expression, *PANDAR* modulates G1/S arrest in breast cancer cells [11]. Additionally, high *PANDAR* levels in colon cancer predict poor patient prognosis and promote metastasis through EMT [12]. lncRNAs also participate in EMT, maintaining the properties of CSCs in breast cancer. Increasing evidence suggests lncRNAs have critical regulatory functions in cancer cell proliferation and apoptosis, cell cycle arrest, metabolism, angiogenesis, invasion, metastasis, and disease relapse [13, 14, 15, 16, 17, 18].

Here, we assessed the effects of *PANDAR* on EMT and proliferation in breast cancer cells.

## Methods

### Cell culture

Human breast invasive ductal carcinoma Michigan cancer foundation 7 (MCF-7) cells were obtained from the American Type Culture Collection (catalog No. HTB-22), HEK-293T cells (catalog No. GNHu17) were obtained from the China Center for Type Culture Collection of the National Collection of Authenticated Cell Cultures Cell Bank affiliated with the Shanghai Institute of Biochemistry and Cell Biology, Chinese Academy of Sciences, and these were maintained in Dulbecco's modified Eagle's medium (DMEM; Gibco) containing 10% fetal bovine serum (Gibco), penicillin (100 U/mL), and streptomycin (100 mg/mL). Cells were incubated in culture medium under a humidified atmosphere of 5% CO_2_ in air at 37 °C. Cell identity had been confirmed by short tandem repeat DNA profiling, and PCR had been used to confirm the absence of any mycoplasma contamination.

### Plasmid construction

The genes of lncRNAs, including *TCONS00068220* (XR_928848.2), which is the same gene as *LOC105375819*, for HOX transcript antisense RNA (*HOTAIR*, NR_003716.3), ciR-has_circ_0001073, *BX647792*, and *PANDAR* (NR_109836.1), were amplified from human genomic DNA isolated from MCF-7 cells. lncRNA were then cloned into the pLVX-Puro vector to construct a series of lncRNA plasmids. Circular RNA ciR-has_circ_0001073 was cloned into pLCDH-ciR vector. Primer sequences are listed as follows: *HOTAIR* forward (F): CAAGCTTCGAATTACGAATTCGACTCGCCTGTGCTCTGGAGCTTG; *HOTAIR* reverse (R): TTATCTAGAGTCGCGGGATCGGAAAATGCATCCAGATATTAATAT; *BX647792* F: CAAGCTTCGAATTACGAATTCGGCCAGAAGGGGAGGAAATTGGAA; *BX647792* R: TTATCTAGAGTCGCGGGATCGCCAGTTTTTCCAACTTCCCCTTTC; *LOC105375819* F: CAAGCTTCGAATTACGAATTCCCGACTTTCACTTATCAGACCTTG; *LOC105375819* R: TTATCTAGAGTCGCGGGATCGGGTGAGCAGTTTTTATTAACCTGT; *PANDAR* F: CAAGCTTCGAATTACGAATTCTTTCAGGAATGCCGCAGATGTACA; *PANDAR* R TTATCTAGAGTCGCGGGATCGCAGTGGCTCACGCCTGTAATCTCA; *ciR-hsa_circ_0001073* F: ACCTCCATAGAAGATTCTAGAGAGTTCTAAAATTAAACTATGTGG; *ciR-hsa_ circ_0001073* R: CTCAGCGCCACAGCAGAATTCGTTAATTGAACAGATTTTATTTTA.

### Dual-luciferase reporter assay

The human and mouse E-cad gene promoters (Nos. 42081 and 61798) reporter plasmids were purchased from Addgene. lncRNA expression vectors, reporter plasmid containing mouse E-cad promoter, and internal control plasmid pRL-TK were cotransfected into HEK-293T cells. The pRL-TK vector served as a reference for normalizing transfection efficiency. At 48 h after transfection, dual-luciferase assays were performed by using a dual-luciferase reporter assay kit (Promega), according to the manufacturer's instructions. The ratio of firefly-to-*Renilla* luciferase activity was calculated and results were normalized to the corresponding negative control. Data are represented as the mean ± standard deviation (SD) of triplicate assays. Significant differences from controls are indicated by *P < 0.05, **P < 0.01.

### Lentivirus packaging and stable cell line construction

LentiX-293 cells were seeded into a 10 cm dish at 2.5 × 10^6^ cells/dish. On the next day, a mixture of 5 μg pLVX-PANDAR or pLVX-Puro, 3.75 μg psPAX2 (catalog No. 12260; Addgene), and 1.25 μg pMD2.G (catalog No. 12259; Addgene) were diluted into 1 mL opti-MEM medium (Invitrogen); then, 30 μL PEI solution (1 mg/mL, catalog No. 23966; Polyscience) was added and mixed thoroughly. The mixture was incubated at room temperature for 25 min to prepare the transfection complexes. Then, the mixture was added dropwise into the abovementioned LentiX-293 cells. At 48 h after transfection, the supernatants were collected and centrifuged at 40,000 × *g* to collect lentivirus pellets. We used 1 mL DMEM medium to resuspend the lentivirus. MCF-7 cells (1 × 10^6^ cells in a 60 mm dish) were infected with each lentivirus at a multiplicity of infection (MOI) of 1. At 48 h after infection, stable cell lines were selected by adding 1 μg/mL puromycin (Sigma).

### RNA extraction and qRT-PCR

Total RNA was extracted from cells using TRIzol reagent (Invitrogen) according to the manufacturer's instructions. The cDNA was synthesized through reverse-transcription reaction with Rever-Tra-Ace-α-Transcriptase (Toyobo) and subsequently amplified by PCR using SYBR Premix Ex Taq II (Tli RNaseH Plus) (TaKaRa Bio). The primer sequences used in quantitative real-time polymerase chain reaction (qRT-PCR) are listed as follows: *GAPDH QRT* F: CCCATGTTCGTCATGGGTGT; *GAPDH QRT* R: TGGTCATGAGTCCTTCCACGATA; *PANDAR* F: TCAGGAATGCCGCAGATGTA; *PANDAR* R: GACCGTGTCTGGAGGATGCC.

### Immunoblotting

The harvested cells were pelleted and resuspended in sodium dodecyl sulfate (SDS) lysis buffer (50 mM pH 6.8 Tris-HCl, 1% SDS, 10% glycerol, and 20 mM dithiothreitol) and incubated on ice. The lysates were centrifuged at 8,000 × *g* (5 min, 4 °C). Subsequently, proteins were separated by 10% SDS-polyacrylamide gel electrophoresis (SDS-PAGE) and electro-transferred onto polyvinylidene difluoride (PVDF) membranes (Millipore, USA). After blocking nonspecific binding sites with 5% nonfat milk for 1 h at ambient room temperature, they were incubated overnight at 4 °C with mouse monoclonal primary antibodies against E-cad (1:1,000; catalog No. sc-21791, antibody ID AB_626777), N-cad (1:1,000; catalog No. sc-59987; antibody ID AB_781744), and β-actin (1:1,000, catalog No. sc-47778; antibody ID AB_2714189) (Santa Cruz Biotechnology). Then, horseradish peroxidase (HRP)-linked goat secondary anti-mouse antibodies (1:2,000; catalog No. A0216; antibody ID AB_2860575; Beyotime, China) were added at ambient room temperature and incubated with the proteins transferred to the membrane for 1 h. Immunoreactive signal was developed and visualized with enhanced chemiluminescence (ECL) reagents (Millipore).

### CCK-8 assay

A cell counting kit-8 (CCK-8) was used to assess cell viability. Briefly, MCF-7 stable cell line overexpressing *PANDAR* or control cells were seeded into 96-well plates at 2 × 10^3^ cells/well. Then, 10 μL CCK-8 reagents (Solarbio, China) were added per well for the indicated time, followed by further incubation at 37 °C for 2–4 h. Optical density was measured at 450 nm on a microplate reader (Bio-Rad).

### Statistical analysis

Data are shown as means ± SD calculated by WPS Office 2019. The empirical data obtained from all experiments were fitted to histograms using GraphPad Prism 7 software. The experiments were performed in triplicate and repeated (more than once). A Student *t* test (two-tailed) was performed to compare parametric data. Differences with *P* < 0.05 were considered significant.

## Results

### *TCONS00068220* and *PANDAR* regulate E-cad gene transcription

**Figure 1:**
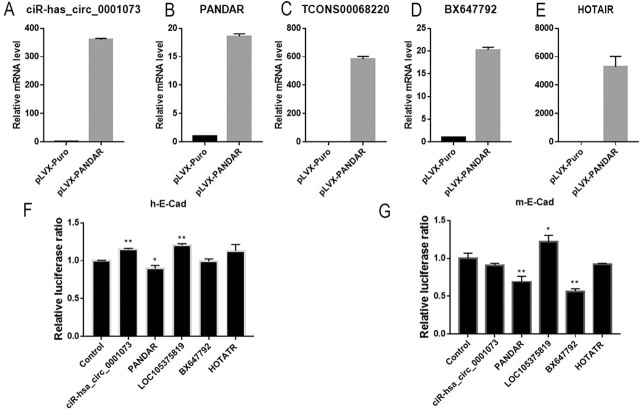
Screening of lncRNAs regulating the activity of the E-cad gene promoter. (**A–E**) The overexpression efficiency of a series of noncoding RNAs were detected by qRT-PCR. (**F, G**) Indicate lncRNA expression vectors, reporter plasmid containing human (**F**) or mouse (**G**) E-cad promoter and the internal control plasmid pRL-TK were cotransfected into HEK-293T cells. We measured luciferase activities 48 h after transfection. The ratio of firefly-to-*Renilla* luciferase activity was calculated and results were normalized to the corresponding negative control. Data are represented as the mean ± SD of triplicate assays. E-cad, E-cadherin; lncRNAs, long noncoding RNAs; SD, standard deviation.

E-cad, a biomarker of epithelial cells, is downregulated in the EMT process. Therefore, lncRNAs altering E-cad expression are involved in carcinogenesis. We constructed a series of noncoding RNA expression vectors and verified their overexpression effects in HEK-293T cells (**Figure 1A–E**). Then, we screened noncoding RNAs that potentially regulated the E-cad gene promoter by dual-luciferase reporter assay, including *HOTAIR*, *LOC105375819*, *BX647792*, and *PANDAR*. The human E-cad promoter reporter plasmid and a series of lncRNAs or circular RNAs were cotransfected into HEK-293T cells. We found that the overexpression of *ciR-hsa_circ_0001073* (1.148 ± 0.014) and *LOC105375819* (1.205 ± 0.019) significantly increased the luciferase activity, whereas overexpression of *PANDAR* (0.897 ± 0.041) suppresses the luciferase activity (**Figure 1F**). This finding suggests that *ciR-hsa_circ_0001073*, *LOC105375819*, and *PANDAR* may regulate E-cad expression in human cells.

To explore which regulation mechanism is evolutionarily conservative, the mouse E-cad promoter reporter vector and a series of lncRNAs or circular RNAs were cotransfected into HEK-293T cells, respectively. This was done to assess whether the abilities of *ciR-hsa_circ_0001073*, *LOC105375819*, and *PANDAR* able to regulate E-cad transcription were evolutionarily conserved. *LOC105375819* (1.219 ± 0.086) remarkably increased luciferase activity, which was reduced by *PANDAR* (0.692 ± 0.071) and *BX647792* (0.566 ± 0.032) (**Figure 1G**). These findings indicated that *LOC105375819* and *PANDAR* regulated the promoter activity of the E-cad gene with evolutionary conservatism.

### *PANDAR* downregulates E-cad, but upregulates N-cad

**Figure 2:**
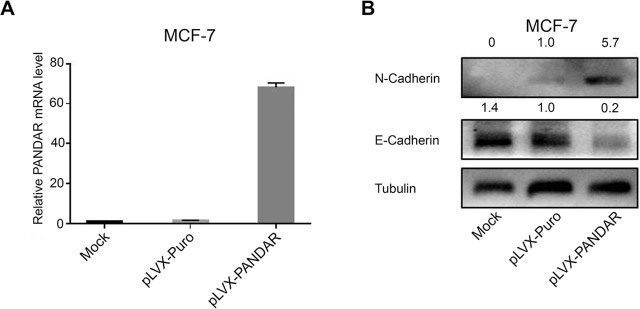
*PANDAR* suppress the expression of E-cad while simultaneously upregulating the expression of N-cad at the protein level. To obtain stable cell lines expressing *PANDAR* and corresponding control, lentiviruses were used to infect MCF-7 cells. (**A**) qRT-PCR were used to determine the overexpression efficiency of *PANAR* in MCF-7 cells. Data are represented as the mean ± SD of triplicate assays. (**B**) Western blotting was performed to determine the expression profile of EMT biomarkers, including E-cad and N-cad protein. Tubulin served as an internal control. E-cad, E-cadherin; EMT, epithelial-mesenchymal transition; MCF-7, Michigan cancer foundation-7; N-cad, N-cadherin; *PANDAR*, promoter of *CDKN1A* antisense DNA damage-activated RNA; SD, standard deviation.

Because *PANDAR* modulated E-cad promoter activity, we hypothesized that it might contribute to modulating the EMT process. First, *PANDAR* was overexpressed in MCF-7 cells by using lentivirus, and then qRT-PCR was used to detect the efficiency of overexpression of *PANDAR* (**Figure 2A**). Further, western blotting was performed to determine the expression profile of EMT-related proteins, including E-cad, and N-cad protein. As depicted in **Figure 2B**, an increased *PANDAR* expression led to elevated N-cad levels. However, it reduced E-cad levels compared with the control group, suggesting that it may potentially control EMT in breast cancer.

### *PANDAR* enhances MCF-7 cell proliferation

**Figure 3:**
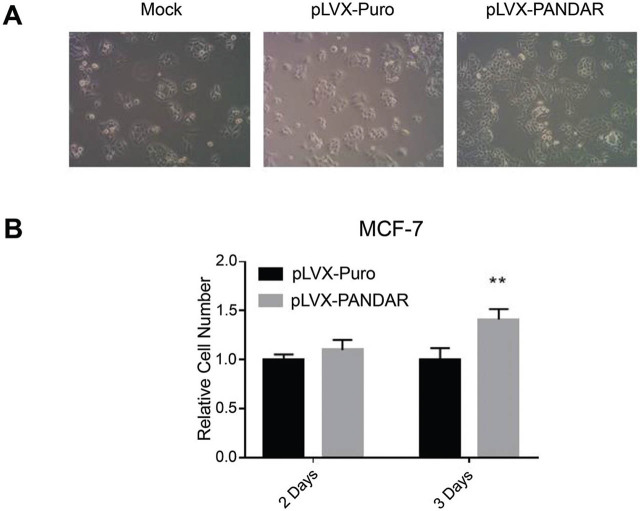
MCF-7 cell proliferation is significantly increased after *PANDAR* overexpression. (**A**) Different treatments of MCF-7 cells were seeded at the same density and photographed under a microscope 3 days later. (**B**) *PANDAR* overexpression in MCF-7 cells through a lentiviral vector. *PANDAR* overexpressing cells and control cells were plated at the same density followed by a CCK-8 assay to quantify cell proliferation. Three days after plating, the MCF-7 cells overexpressing *PANDAR* had proliferated significantly. Each experiment was repeated 4 times. Data are represented as the mean ± SD. CCK-8, cell counting kit-8; MCF-7, Michigan cancer foundation-7; *PANDAR*, promoter of *CDKN1A* antisense DNA damage-activated RNA; SD, standard deviation.

MCF-7 cell expressing *PANDAR* or corresponding control was seeded into 96-well plates with the same density, followed by the photomicrographs and CCK8 assay to quantitate cell proliferation at indicated times (**Figure 3A, B**). The overexpression of *PANDAR* significantly increased MCF-7 cell proliferation at day 3 post-plating (1.000 ± 0.120 vs 1.412 ± 0.105). This significant increase in MCF-7 cell proliferation suggests that *PANDAR* is an oncogene regulating breast cancer proliferation.

## Discussion

Several previously constructed recombinant lncRNA plasmids were screened by dual-luciferase reporter assay. In the present research, *PANDAR*, *LOC105375819*, *HOTAIR*, *ciR-has_circ_0001073*, *BX647792*, and *PANDAR* were compared with the control group. The results revealed that *LOC105375819* significantly promoted the transcription of the *E-cad* promoter, whereas *PANDAR* significantly inhibited the transcription of the *E-cad* promoter. Indeed, it was previously reported that high expression of *PANDAR* in colon cancer predicts poor patient prognosis and promotes metastasis through EMT [12]. The regulatory mechanism of lncRNA on the promoter is more complicated, and most of the regulatory effects belong to fine-tuning. Some lncRNAs can regulate gene expression by adsorbing endogenous miRNAs. Some lncRNAs can be used as scaffolds to bind to promoters to recruit other transcription factors. The molecular mechanism of *PANDAR* regulating E-cad remains unclear. The weak regulatory effect of *PANDAR* on *E-cad* promoters may imply an indirect regulation.

**Table 1:** Primer sequences.

	**Upstream primer sequence (5′–3′)**	**Downstream primer sequence (5′–3′)**
HOTAIR	CAAGCTTCGAATTACGAATTCGACTCGCCTGTGCTCTGGAGCTTG	TTATCTAGAGTCGCGGGATCGGAAAATGCATCCAGATATTAATAT
TCONS00068220	CAAGCTTCGAATTACGAATTCCCGACTTTCACTTATCAGACCTTG	TTATCTAGAGTCGCGGGATCGGGTGAGCAGTTTTTATTAACCTGT
PANDAR	CAAGCTTCGAATTACGAATTCTTTCAGGAATGCCGCAGATGTACA	TTATCTAGAGTCGCGGGATCGCAGTGGCTCACGCCTGTAATCTCA

Western blotting was performed to determine the expression profile of EMT-related proteins, including E-cad, and N-cad. The results show that *PANDAR* inhibited the expression of the E-cad. The core promoter region of the E-cad gene derived from humans differs considerably from that found in mice. However, the human gene for PANDAR also regulated the *E-cad* promoter of mice, which further implies that the regulatory effect of *PANDAR* on the *E-cad* promoter may depend on a small number of nucleic acids or a spatial structure of the promoter that is evolutionarily conserved.

By contrast, *PANDAR* was overexpressed in MCF-7 cells through lentiviral infection, followed by the CCK8 assay to quantify cell proliferation. The CCK-8 assay also established that *PANDAR* upregulation markedly enhanced proliferation of MCF-7 cells. A previous study revealed that *PANDAR* is activated in a p53-dependent fashion and interacts with nuclear transcription factor YA (NF-YA) to suppress proapoptotic genes, thereby inhibiting cell apoptosis [19]. Additionally, *PANDAR* plays an oncogenic role in bladder cancer by inducing cell proliferation and suppressing proapoptotic pathways [20]. *PANDAR* was demonstrated to modulate G1/S arrest in breast cancer cells via p16^INK4A^ downregulation [11]. These data are consistent with our results showing that *PANDAR* promotes breast cancer cell proliferation.

## Conclusion

The lncRNA *PANDAR* regulates the inhibition of the promoter activity of the E-cad gene and promotes breast cancer cell proliferation. Although further functional experiments are necessary, the current findings provide new insights into the molecular mechanisms underpinning *PANDAR*'s effect on the EMT process in breast cancer.
